# Eml1 loss impairs apical progenitor spindle length and soma shape in the developing cerebral cortex

**DOI:** 10.1038/s41598-017-15253-4

**Published:** 2017-12-11

**Authors:** Sara Bizzotto, Ana Uzquiano, Florent Dingli, Dmitry Ershov, Anne Houllier, Guillaume Arras, Mark Richards, Damarys Loew, Nicolas Minc, Alexandre Croquelois, Anne Houdusse, Fiona Francis

**Affiliations:** 1 INSERM UMR-S 839, 17 rue du Fer à Moulin, Paris, 75005 France; 2 Sorbonne Universités, Université Pierre et Marie Curie, 4 Place Jussieu, Paris, 75005 France; 3 Institut du Fer à Moulin, 17 rue du Fer à Moulin, Paris, 75005 France; 4 Institut Curie, PSL Research University, Centre de Recherche, Laboratoire de Spectrométrie de Masse Protéomique, 26 rue d’Ulm, 75248 Cedex 05 Paris, France; 5 Institut Jacques Monod, UMR7592 CNRS Paris, France; 6 Department of Biochemistry, University of Leicester, Henry Wellcome Building, Lancaster Road, Leicester, LE1 9HN UK; 7 Department of Clinical Neuroscience, Centre Hospitalier Universitaire Vaudois and University of Lausanne, 21 rue du Bugnon, 1011 Lausanne, Switzerland; 8 Department of Fundamental Neurosciences, University of Lausanne, 1005 Lausanne, Switzerland; 9 Structural Motility, Institut Curie, Centre de Recherche; CNRS, UMR144, 26 rue d’Ulm, Cedex 05, Paris, 75248 France; 10 Departments of Pediatrics and Neurology, Harvard Medical School, Boston, MA USA; 11 Broad Institute of MIT and Harvard, Cambridge, MA USA; 12 Division of Genetics and Genomics, Manton Center for Orphan Disease, and Howard Hughes Medical Institute, Boston Children’s Hospital, Boston, MA USA

## Abstract

The ventricular zone (VZ) of the developing cerebral cortex is a pseudostratified epithelium that contains progenitors undergoing precisely regulated divisions at its most apical side, the ventricular lining (VL). Mitotic perturbations can contribute to pathological mechanisms leading to cortical malformations. The *HeCo* mutant mouse exhibits subcortical band heterotopia (SBH), likely to be initiated by progenitor delamination from the VZ early during corticogenesis. The causes for this are however, currently unknown. Eml1, a microtubule (MT)-associated protein of the EMAP family, is impaired in these mice. We first show that MT dynamics are perturbed in mutant progenitor cells *in vitro*. These may influence interphase and mitotic MT mechanisms and indeed, centrosome and primary cilia were altered and spindles were found to be abnormally long in *HeCo* progenitors. Consistently, MT and spindle length regulators were identified in EML1 pulldowns from embryonic brain extracts. Finally, we found that mitotic cell shape is also abnormal in the mutant VZ. These previously unidentified VZ characteristics suggest altered cell constraints which may contribute to cell delamination.

## Introduction

The mammalian cerebral cortex develops from neural progenitors that form a specialized proliferative layer in the developing brain, the VZ. Radial glial cells (RGCs), also named apical progenitors (APs), are the most abundant cells that divide in this zone, and are able to both self-renew and to produce other cell types, being crucial for post-mitotic neuron development in the cortex. These cells have a specialized morphology with apical and basal processes that anchor them to the VL and pial surface respectively^1^. In interphase RGCs, centrosomes are located at the extremity of apical processes and delineate the VL. The centrosome is tightly connected with the primary cilium, which is also localized in apical end-feet of RGCs during interphase. The primary cilium is an MT-based organelle, which projects towards the ventricle in order to sense signals from the cerebrospinal fluid^2^. RGC nuclei move apico-basally during the cell cycle in a characteristic process known as interkinetic nuclear migration (INM). Mitosis occurs when the nuclei are in contact with the VL. Prior to mitosis, centrosomes move a short distance basally before undergoing duplication and forming the spindle poles^3^. Ciliary remnants keep in close contact with the mother centriole and may play a role in daughter cell fate^4^. The importance of correctly regulated RGC morphology and division is indicated by the numerous cortical malformation phenotypes observed in mouse mutants with a perturbed VZ^5^.

We focus here on the spontaneous *HeCo* mouse mutant^6,7^, which shows heterotopia and a proportion of abnormal RGCs dividing outside the VZ during development^7^. Ectopic proliferating cells expressing RGC markers are found in the intermediate zone (IZ) and cortical plate (CP) at embryonic day (E) 13.5, coincident with early-mid corticogenesis, which supports the idea that delamination of a proportion of cells from the VZ might be the primary cause of the heterotopia phenotype^7^. The mechanisms responsible for delamination, which occur in a number of mouse mutants and physiologically in primate and human brains^8,9^, are however still unclear and the focus of intense interest. Apical cell junction markers do not appear to be majorly disrupted in the *HeCo* VL^7^ which has been shown to be a sign of RGC abnormalities in other mutants^10,11^.

The mutant gene in *HeCo* mice is *Eml1* (*E* *chinoderm microtubule-associated protein like-1*, also known as *Emapl*-1), coding for a MT-binding protein expressed in the VZ during cortical development^7^ and whose function in APs is unknown. The mutation found in *HeCo Eml1* consists of the insertion of a retrotransposon in the last intron of the gene, leading to the absence of the full-length transcript and protein^7^. We showed previously that recombinant Eml1 binds directly to MTs *in vitro* and strongly co-localizes with the MT network during both interphase and mitosis in progenitor cells^7^. Several other members of the EMAP family, such as sea urchin EMAP, Xenopus XMAP, and mammalian EML2, EML3 and EML4, also participate in the regulation of MT dynamics, including within the mitotic spindle^12–16^. However, this family of proteins, and especially EML1, remains poorly studied.

Regulation of the spindle is a finely controlled process, and mutations have been found in several spindle genes which severely disrupt the formation of the cortex^5^. In a given cell type, the steady-state metaphase spindle is characterized by constant pole-to-pole spacing^17^, which is determined by the balance between intrinsic factors influencing MT dynamics and assembly, as well as cell boundary constraints^17–22^. The correct interplay between metaphase spindle length, cell size and shape is important for the accurate positioning of the spindle within the cell, which influences chromosome segregation and selection of the cell division plane^23–25^. This is known to be critical for correct cortical development. In the *HeCo* developing telencephalon, AP mitotic spindles were found to have a significantly increased percentage of oblique cleavage planes at anaphase^7^. The causes of this phenotype are not yet known.

Cells change their size, shape, number and position during development, which is fundamental for proper tissue morphogenesis^26,27^. These cell properties have up till now been little studied in the neuroepithelium of the developing cortex. Developmental changes in the VZ require APs like other epithelial cells, to respond to surrounding mechanical forces^28,29^. It is also well-known that intrinsic cellular characteristics, as well as space constraints can together control cell proliferation^30^. INM has been proposed to generate space for mitosis^29^, occurring as nuclei reach the VL. Factors, which are as yet little-known, must regulate spindle assembly and orientation, symmetric versus asymmetric division, as well as daughter cell attachment or detachment. It seems important then to consider features of the mitotic spindle, as well as cell size and shape to learn more about these processes.

Focusing on *HeCo* mice, showing gene mutations in the MT-binding protein Eml1, we show altered MT dynamics in mutant cells compared to wild-type (WT) *in vitro*. In E13.5 APs in brain slices, centrosomes and primary cilia are perturbed and there are abnormal prometaphase/metaphase ((pro)metaphase) spindle lengths. We also assessed cell shape and density in this region and show that (pro)metaphase cells dividing at the VL have abnormal shapes in the mutant brain. This work thus defines novel pathological VZ characteristics that may contribute to the delamination of a proportion of APs during early-mid corticogenesis.

## Results

### MT dynamics are perturbed in *HeCo* progenitors *in vitro*

**Figure 1:**
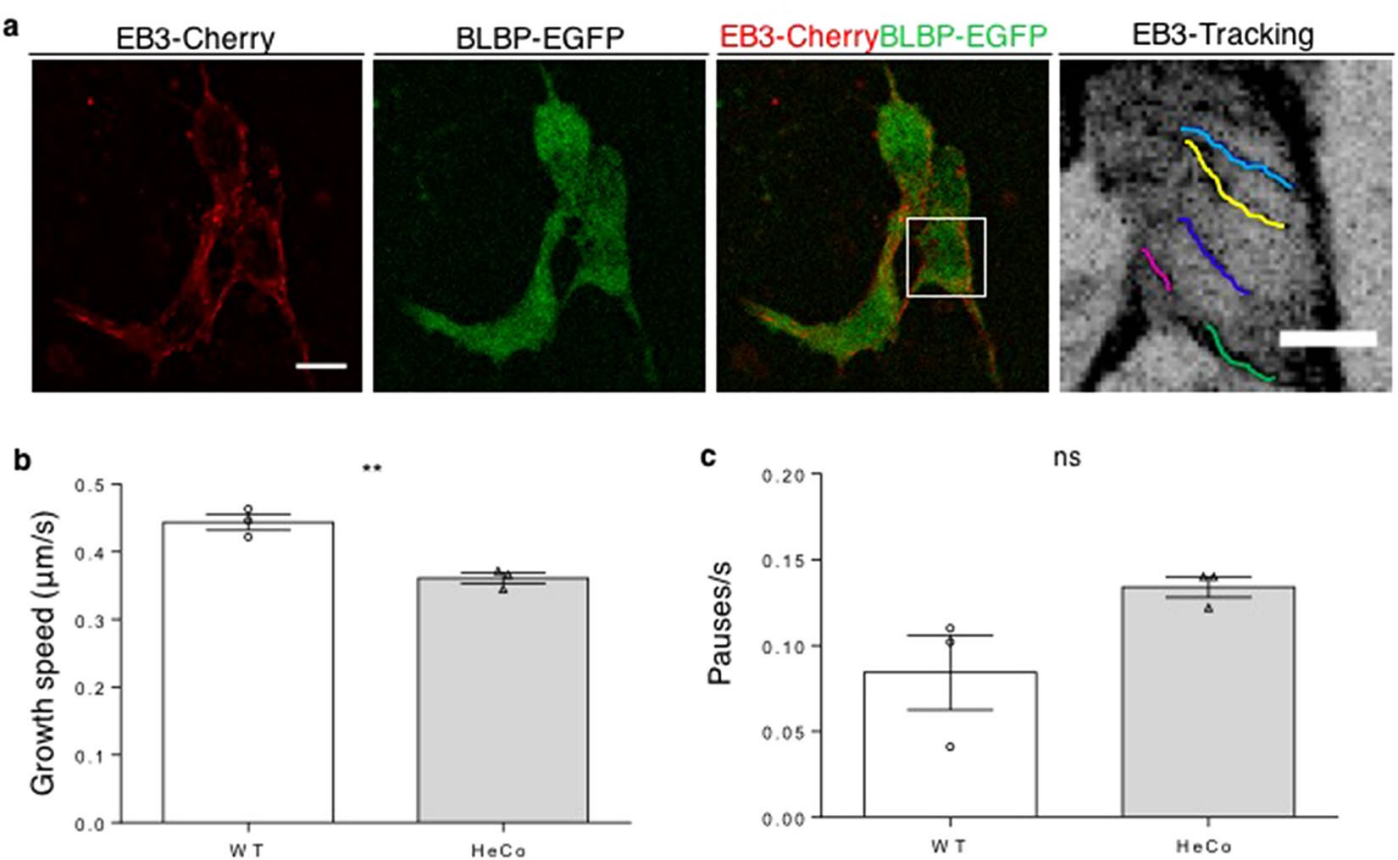
*HeCo* progenitors show perturbed MT dynamics. (**a**) Cultured dissociated neural progenitors from E12.5 mouse cortices transfected with EB3-mCherry (red) and BLBP-EGFP (green). Interphase BLBP + cells were imaged to assess EB3 tracking. Each colored line in the higher magnification (right) shows one EB3 track. (**b**) EB3-labelled MT plus-ends advance more slowly in *HeCo* cells compared to WT. Each dot (WT) or triangle (*HeCo*) represents a different experiment. Average track speed for the two conditions is shown by bars. (**c**) EB3-labelled MT plus-ends do not show significantly increased pause frequencies in *HeCo* progenitors. N = 3, 198 total tracks from 23 cells measured for WT, 180 total tracks from 17 cells measured for *HeCo*. Unpaired *t*-test. **P < 0.01, ns = non-significant. Scale bars, 10 μm (**a**, lower magnification), 5 μm (**a**, higher magnification).

Recombinant Eml1 was initially shown to be enriched at the MT-organizing center (MTOC) and potentially associated with polymerizing MTs in non-neuronal interphase cells in culture^7^. A co-localization was also shown with MTs in Pax6-positive (+) progenitors *in vitro* ^7^. Here we tested MT growth in mutant neural progenitors in culture by measuring the plus-end elongation rate by live-imaging. Primary cultures enriched for neural progenitors were prepared from embryonic WT and *HeCo* cortices dissected at E12.5, a time-point when Eml1 is already expressed in the VZ (Supplementary Fig. S1a). These were co-transfected with plasmids expressing fluorescent plus end-binding (EB)3-mCherry protein and Enhanced Green Fluorescent Protein (EGFP), the latter under the control of the brain lipid-binding protein (BLBP) promoter^31^ which is specifically active in RGCs. Cells were then analyzed at two days *in vitro* (DIV), allowing for expression of fluorescent proteins (Fig. 1a), and at a time-point approximately equivalent to the developmental stage E13.5-E14.5 *in vivo*. Clusters of EGFP + interphase cells were live-imaged for EB3-mCherry (Supplementary Video S1), and the movies obtained analyzed to track EB3 movement (Supplementary Video S2). The growth speed of single MTs was lower in *HeCo* progenitors, compared to WT cells (Fig. 1b). We compared the frequency of stalling during MT growth and although a tendency for increased pausing was possible, the differences were not significant between WT and *HeCo* (Fig. 1c). Thus, interphase *HeCo* mutant cells in culture have perturbed MT plus-end growth dynamics compared to WT, mostly associated with a decreased polymerization rate.

We attempted to rescue the MT dynamic phenotype by reintroducing Eml1 in cultured *HeCo* progenitors through transfection of a construct expressing both Eml1 and EGFP, under the control of the BLBP promoter (pBLBP-Eml1-IRES-EGFP)^7^. However, consistent with an Eml1 overexpression phenotype also decreasing MT polymerization observed in Neuroblastoma-2A (N2A) cells (Supplementary Fig. S1b–d), MT growth rate was still lower in EGFP + *HeCo* progenitors after Eml1 transfection, showing no significant difference compared to mutant progenitors transfected with the control vector pBLBP-EGFP (Supplementary Fig. S1e,f). *HeCo* progenitors transfected with Eml1 also showed an increase in the frequency of stalling during the polymerization. A similar tendency was also observed upon Eml1 overexpression in N2A cells. Comparing growth rates without taking into account the pauses still showed significantly reduced growth rates in both cell types (Supplementary Fig. S1g,h). Thus, these combined results suggest that MT growth rate is altered in *HeCo* progenitors and that Eml1 is crucial for MT dynamics, and either an absence or overexpression of the protein reduces MT growth rate. Eml1 re-expression in mutant cells under these conditions was not therefore able to rescue the phenotype.

### Centrosome and primary cilia defects, as well as abnormally long (pro)metaphase spindles in the *HeCo* VZ at E13.5

**Figure 2:**
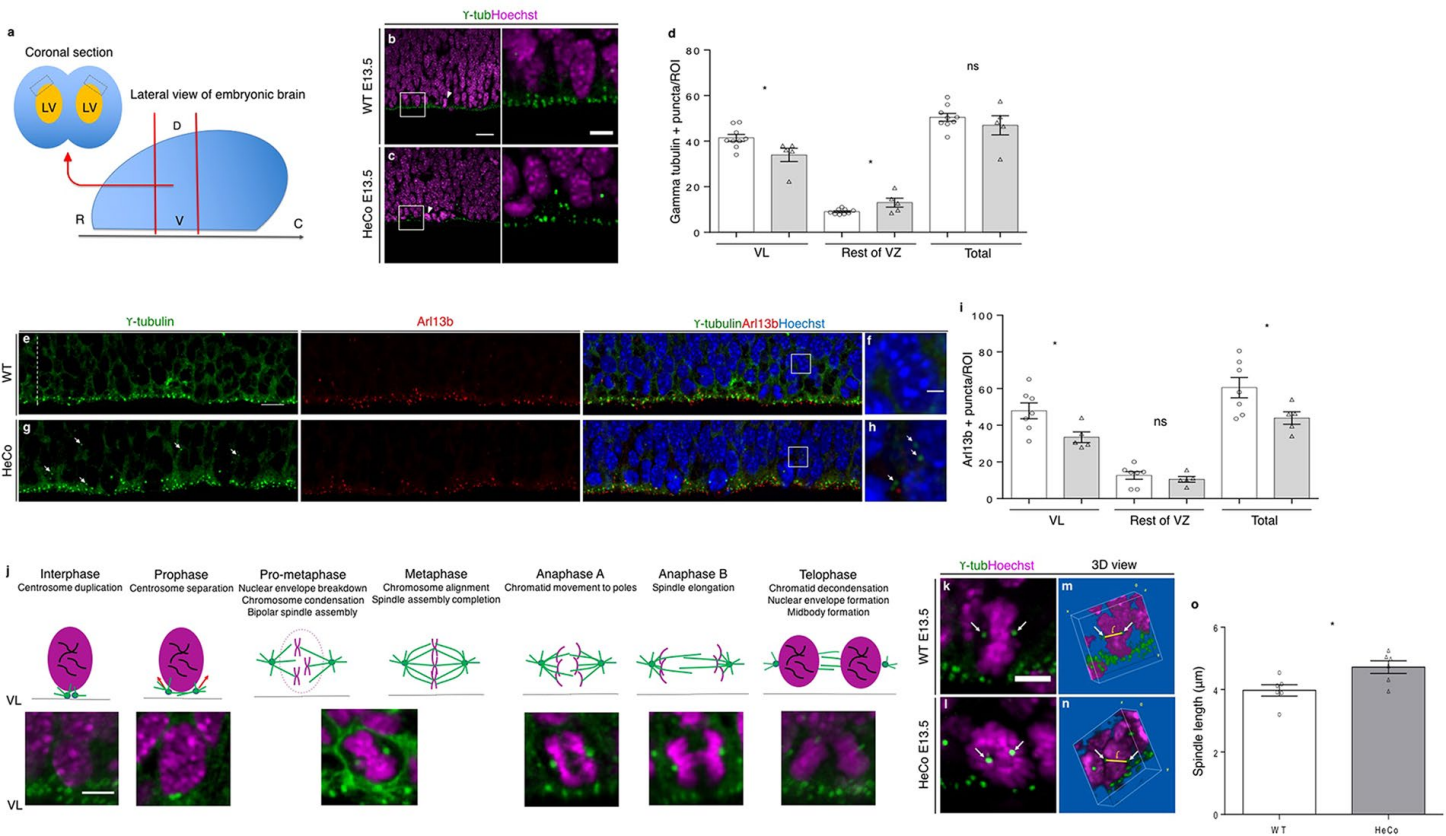
VL anomalies and abnormal (pro)metaphase spindle length in the *HeCo* VZ. (**a**) Coronal sections corresponding to the region delimited by red lines were immunostained and the VL analyzed (grey boxes top left). (**b**,**c**) γ-tubulin (green) and Hoechst (magenta) staining in WT (**b**) and *HeCo* (**c**) E13.5 brain sections. Higher magnifications show perturbed γ-tubulin staining in *HeCo* brains. (**d**) Less centrosomes in the *HeCo* VL and increased number in more basal positions. The total number of VZ centrosomes does not change between WT and *HeCo*. (**e–h**) γ-tubulin (green), Arl13b (red) and Hoechst (blue) staining in E13.5 WT and *HeCo* VZ. Small white line indicates the VL. Dashed white line indicates the rest of the VZ (3-soma height). Higher magnifications (**f,h**) show mislocalized basal centrosomes (white arrows) not associated with primary cilia in *HeCo*. (**i**) Less Arl13b + puncta in the *HeCo* VL but no change in the rest of the VZ. Overall, less primary cilia in the *HeCo* VZ compared to WT. (**j**) Mitotic phases and corresponding images from APs in brain slices. Purple, nuclei and chromosomes; green dots, centrosomes; green lines, MTs. Pro-metaphase and metaphase are not distinguishable from our images. (**k**,**l**) Isolated single (pro)metaphase cells. Spindle poles labelled by γ-tubulin (white arrows) are visible. (**m**,**n**) 3D reconstructions illustrating pole-to-pole distance measurements. Yellow bars indicate the pole-to-pole (white arrows) distance (l) measured. Only (pro)metaphase cells located at the VL were measured. (**o**) Spindles are longer in *HeCo* brains at E13.5 compared to WT. Circles (WT) and triangles (*HeCo*) represent single embryos. Average for each condition is represented by bars. γ-tubulin: WT, N = 9 embryos from 3 litters; *HeCo*, N = 5 embryos from 3 litters. Arl13b: WT, N = 7 embryos from 5 litters; *HeCo*, N = 5 embryos from 4 litters. Spindle length: N = 6 embryos from 3 litters for each condition. WT, 176 cells; *HeCo*, 160 cells. Unpaired *t*-test. *P < 0.05, ns = non-significant. Scale bars, 20 µm (**b,e**), 10 µm (**e,g**), 5 µm (higher magnification in **b**,**j,k**) and 2 µm (higher magnification **f**). R, rostral; C, caudal; V, ventral; D, dorsal; LV, lateral ventricle; VL, ventricular lining; VZ, ventricular zone.

MT dynamics are critical for several aspects of the cell cycle^32–35^. We analyzed APs in the VZ directly in dorso-medial WT and *HeCo* developing cortex (Fig. 2a), analyzing cells in their 3D tissue environment. We performed this at E13.5, when *Eml*1 is expressed in the VZ, and a proportion of ectopic progenitors are already identifiable in the mutant cortex^7^. In WT VZ, γ-tubulin staining revealed apical well-aligned centrosomes (Fig. 2b), and more basally located centrosomes associated with dividing cells. In *HeCo* brains, γ-tubulin revealed a more disorganized staining (Fig. 2c). We quantified the number of γ-tubulin + puncta at the VL (3 µm thick), and above in a three-soma height within the VZ, and found that *HeCo* VLs contained significantly less puncta compared to WT (Fig. 2d). In addition, more γ-tubulin + puncta were present above the VL, not obviously associated with dividing cells. Overall, the total number of centrosomes did not differ between WT and *HeCo*, suggesting a changed position of some centrosomes toward more basal positions in the mutant.

Because centrosomes are closely related to primary cilia in RGC apical processes^4^, we also performed a co-staining between γ-tubulin and ADP ribosylation factor like GTPase 13b (Arl13b), a primary cilium marker, to check their mutual localization in WT versus *HeCo* VZ (Fig. 2e–h). We quantified the number of Arl13b + puncta again at the VL and in a 3-soma height of the VZ (Fig. 2i). A reduction in the number of Arl13b-puncta was observed in the *HeCo* VL but the rest of the VZ showed similar numbers compared to WT. Overall, the total number of puncta was reduced in the mutant. Thus, as confirmed by our images, the excess of basally-localized γ-tubulin puncta are often not associated with Arl13b (Fig. 2h), and primary cilia numbers appear reduced at the VL of *HeCo* mice. These results show different behaviors of two highly associated organelles in APs, potentially indicating apical end-foot detachment and/or VL anomalies.

We next focused on spindle length, a parameter that is finely regulated by MT dynamics and previously unexplored in APs^17^. Hoechst staining, together with γ-tubulin, was used to identify different phases of the cell cycle. WT and mutant metaphase and pro-metaphase cells appeared indistinguishable in number and aspect, and there are no indications of delayed mitosis in *HeCo* APs^7^. The resolution of images, together with 3D reconstruction of confocal z-stacks corresponding to cropped single cells at the dorso-medial VL (see Materials and Methods for more details on the analysis), allowed us to distinguish the different mitotic phases (Fig. 2j), although it was not possible based on DNA shape to firmly discriminate between pro-metaphase and metaphase. γ-tubulin was used to identify the separated spindle poles when they were located at the opposite sides of the metaphase plate, and allowed individual pole-to-pole distances to be measured in (pro)metaphase cells, which we referred to as spindle lengths (Fig. 2k–n). These are likely to be maximal (metaphase) or near-maximal (pro-metaphase). Remarkably, average spindle length was found to be longer in E13.5 *HeCo* APs compared to WT (Fig. 2o). We also checked whether longer (pro)metaphase spindles were associated with oblique/horizontal cleavage planes (metaphase plate orientation with respect to the VL) shown to be increased in mutant APs^7^. However, we found that spindle lengths during (pro)metaphase were on average the same for vertical or non-vertical cleavage planes (Supplementary Fig. S2a), which suggests that spindle length in (pro)metaphase APs is not a predictor of spindle orientation and *vice versa*.

We also assessed whether spindle length was altered in cells dividing in more basal positions, away from the VL (non-VL). Basally positioned (pro)metaphase cells were identified in *HeCo* cortices by their location as well as DNA condensation, as previously performed for the VZ. We found that these cells had spindle lengths which were comparable to *HeCo* VL cells (Supplementary Fig. S2b). Similarly, a tendency for longer mutant spindles was also revealed when directly comparing basal (pro)metaphase WT to *HeCo* basally dividing cells (Supplementary Fig. S2c). Thus, as well as centrosome and primary cilia defects, our results show a defect in pro(metaphase) spindle pole-to-pole distance at early-mid corticogenesis in the mutant.

### Eml1 MT-related protein partners in E13.5 cortices

**Figure 3:**
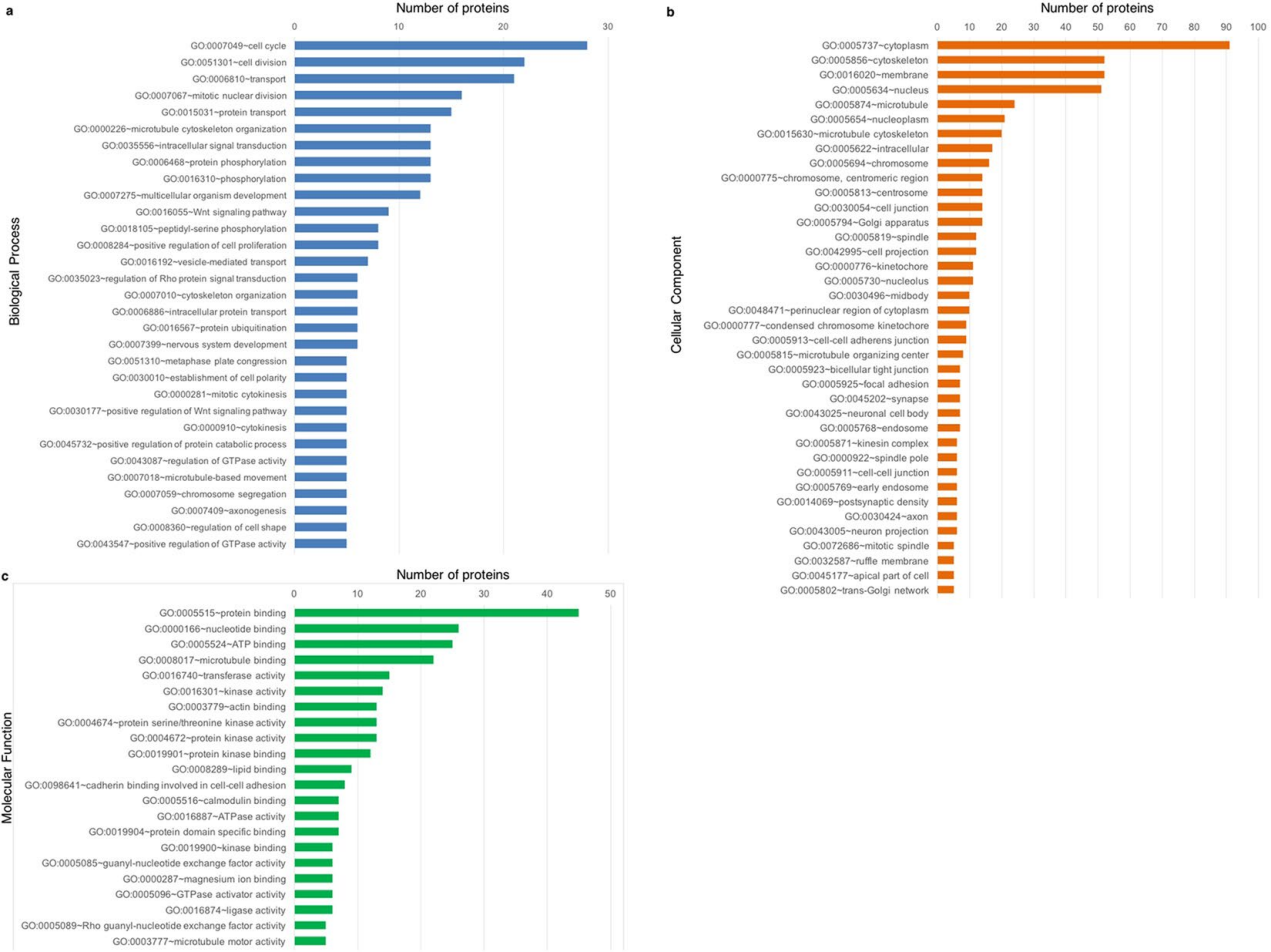
MS analyses reveal EML1 N-ter interactors. (**a**–**c**) GO analyses for the filtered 176 protein list performed with the DAVID Functional Annotation Tool. Classified proteins are specific for EML1 Nter based label-free quantification analysis. GOs based on biological process (**a**), cellular component (**b**), and molecular function (**c**) were generated.

To identify molecular partners of Eml1 in embryonic brain, we performed pull down experiments from mouse E13.5 cortices. Structural studies showed that the isolated EML1 N-terminal domain (N-ter, amino acids 1–174, 91% identity with mouse Eml1) contains a coiled-coil region, mediates homo-trimerization, and binds MTs^36,37^. The larger C terminal domain contains WD40 repeats making up tandem beta-propeller structures^36^. We searched for protein partners potentially influencing MT association, since two of the missense mutations identified in patients may directly or indirectly affect this function (R138X disrupting N-ter, and T243A, previously shown to sediment less with MTs^7^). Pull-down experiments were performed with purified glutathione-S-transferase (GST)-tagged EML1 N-ter and E13.5 WT embryonic cortex extracts (Supplementary Fig. S3). Samples were analyzed by mass spectrometry (MS) to identify direct and/or indirect partners of the protein. Label-free quantitative analyses based on the extracted ion chromatogram (XIC) method, comparing GST-Nter EML1 to GST control samples, revealed a list of 1059 proteins (listed in Supplementary Table S1) uniquely associated with EML1 N-ter. This list was further filtered to exclude proteins often found abundant in MS analyses and therefore less likely to be specific partners of Eml1, such as histones and RNA-processing proteins. Nuclear proteins associated with nucleic acids (e.g. transcription factors), and extracellular matrix proteins were also excluded since Eml1 is found primarily in the cytoplasmic compartment. A new list of 176 proteins was obtained after filtering (Supplementary Table S2). Gene ontology (GO) analyses were performed using the Database for Annotation, Visualization and Integrated Discovery (DAVID) Functional Annotation Tool^38^ (https://david.ncifcrf.gov/home.jsp) (Fig. 3a–c, also see Supplementary Table S2). The most represented biological processes were cell cycle, cell division and transport (Fig. 3a). Concerning cellular component, proteins were classified mainly as cytoplasmic, as expected based on our exclusion criteria. Among these, many proteins were found in the cytoskeleton and membrane categories (Fig. 3b). Molecular function classification identified the majority of proteins as having protein binding activity, and nucleotide, ATP and MT binding were well represented (Fig. 3c).

**Figure 4:**
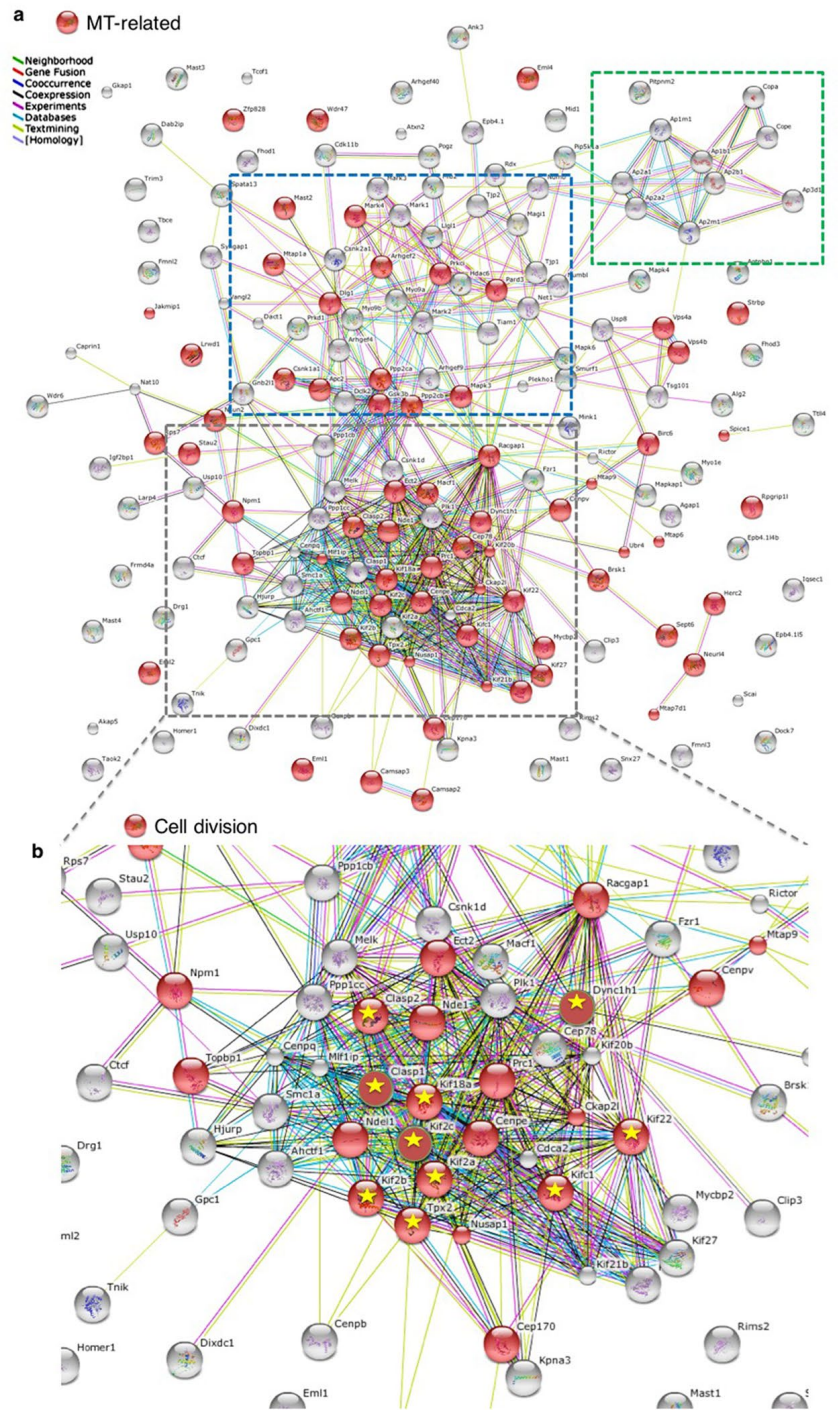
EML1 N-ter interactors are involved in cell division, MT processes and spindle length regulation. (**a**) STRING functional protein association network performed on the 176 protein list. Red items are proteins associated with the MT cytoskeleton based on STRING GO classification which contains functional categories as defined for the Clusters of Orthologous Groups (COG) database. *P*-value < 0.05. Grey, blue and green dotted lines indicate highly interconnected protein clusters. (**b**) The network boxed in grey in A is shown in higher magnification. Red items are proteins associated with the mitotic spindle based on STRING GO classification. *P*-value < 0.05. Proteins marked by yellow stars are associated with spindle length regulation based on literature searches (see also Table 1).

The 176 list was then analyzed using the Search Tool for the Retrieval of Interacting Genes/Proteins (STRING) Functional Protein Association Network^39^ (string-db.org) based on known and predicted protein-protein interactions (Fig. 4). The software was able to connect the majority of items, with only a small percentage of disconnected nodes. A large network of highly interacting nodes was revealed (grey box in Fig. 4a). This contained several proteins already known to be mutated in cortical malformations and related to neural progenitor function, such as kinesin superfamily protein (Kif) 20b, Kif2A, polo kinase 1 (Plk1), nuclear distribution E neurodevelopment protein 1 (Nde1), Nde1-like (Ndel1) and dynein heavy chain 1 (Dync1h1)^40–42^. Two additional smaller clusters were also obvious: one (blue box in Fig. 4a) containing mostly protein kinases and phosphatases involved in the regulation of actin and MT cytoskeletons; a smaller cluster (green box in Fig. 4a) contained proteins involved in vesicle coating and intracellular transport.

We confirmed by the STRING GO tool that a high proportion of proteins was related to the MT cytoskeleton (red items in Fig. 4a), and this was especially true for the highly interacting nodes. Eml1, Eml2 and Eml4 were identified amongst these proteins revealing probable heteromerization of these Emls in brain developmental cells. In the major STRING cluster (grey box), half of the nodes were associated with cell division (Fig. 4b, red nodes) and amongst these 15 molecules had motor activity, 10 being kinesins (listed in Supplementary Table S2). Notably as well, among the interconnected mitotic spindle proteins, a number^10^ are known already to regulate spindle length (yellow stars in Fig. 4b; see also Table 1). Using publicly available resources (http://www.genepaint.org), *Kif2*C and *Tpx*2 (*targeting protein for Xklp*2) *in situ* hybridization also showed strong expression in the VZ (Supplementary Fig. S4a), and *KifC*1, *Kif2*A, *Kif*22 and *Dync*1h1 showed expression in both the VZ and CP, similar to *Eml*1 at the same age^7^ (Supplementary Fig. S4a). In addition, to assess if Eml1-interacting proteins present a different pattern of expression in *HeCo* brains compared to WT, we performed immunohistochemistry (IHC) for some selected partners: Kif22, Kif18A and Dync1h1. Notably, the MT motor protein Kif22 showed a different pattern of expression in *HeCo* cortices. Compared to WT, where Kif22 seems to be relatively concentrated at the VL, and localized to a lesser extent basally in the VZ, Kif22 staining was more obvious throughout the VZ in the mutant, with a relative reduced intensity at the apical surface (Supplementary Fig. S4b). In these preliminary IHC studies, no differences were observed for Kif18A and Dync1h1 (Supplementary Fig. S4c,d).

**Table 1:** Spindle length regulators identified by MS analysis for EML1 N-ter, and summary of their effects on MT dynamics and spindle length.

Protein	Action on MTs	Inactivation effect on spindle length	Expression in the VZ during corticogenesis	Reference
KifC1 (kinesin-14)	Cross-linking and bundling of parallel MTs (minus-end directed)	Shorter spindles	Yes	17,62
Kif22 (kinesin-10)	Cross-linking and bundling of parallel MTs (plus-end directed)	Shorter spindles?	Yes	17,54
Kif18A (kinesin-8)	Plus-end capping motor that halt MT growth	Longer spindles	?	63
Kif2A (kinesin-13)	MT depolymerization (minus-end)	Longer spindles	Yes	64,65
Kif2B (kinesin-13)	MT depolymerization	?	?	64,65
Kif2C (kinesin-13)	MT depolymerization (plus-end)	Longer spindles	Yes	64,65
Kif10 (Cenp-E)	MT stabilization (through binding to CLASPs)	?	?	66
Tpx2	MT bundling and nucleation	Shorter spindles	Yes	67
Clasp1	MT stabilization (plus-end)	Shorter spindles	?	68,69
Clasp2	MT stabilization (plus-end)	Shorter spindles	Yes	68,69
Dync1h1	Minus-end directed motor	Longer spindles	Yes	17
Eml4	Overexpression decreases MT growth rate	?	Yes	15
Eml1	Absence of functional Eml1 leads to slower MT plus-end growth	Longer in E13.5	Yes	This study

Proteins are indicated that give rise to shorter or longer spindles following inactivation that give rise to shorter or longer spindles following inactivation. Known expression in the VZ during mouse cortical development is reported (see also Supplementary Figure S4a). Eml1 is also shown in the table for comparison.

Thus, in fitting with the perturbed length of *HeCo* spindles, MS analyses for Eml1 partners identify a tight network of proteins, many of which are involved in MT function and spindle length regulation, as well as centrosome behavior.

### (Pro)metaphase APs show abnormal soma shape in *HeCo* brains at early-mid corticogenesis

**Figure 5:**
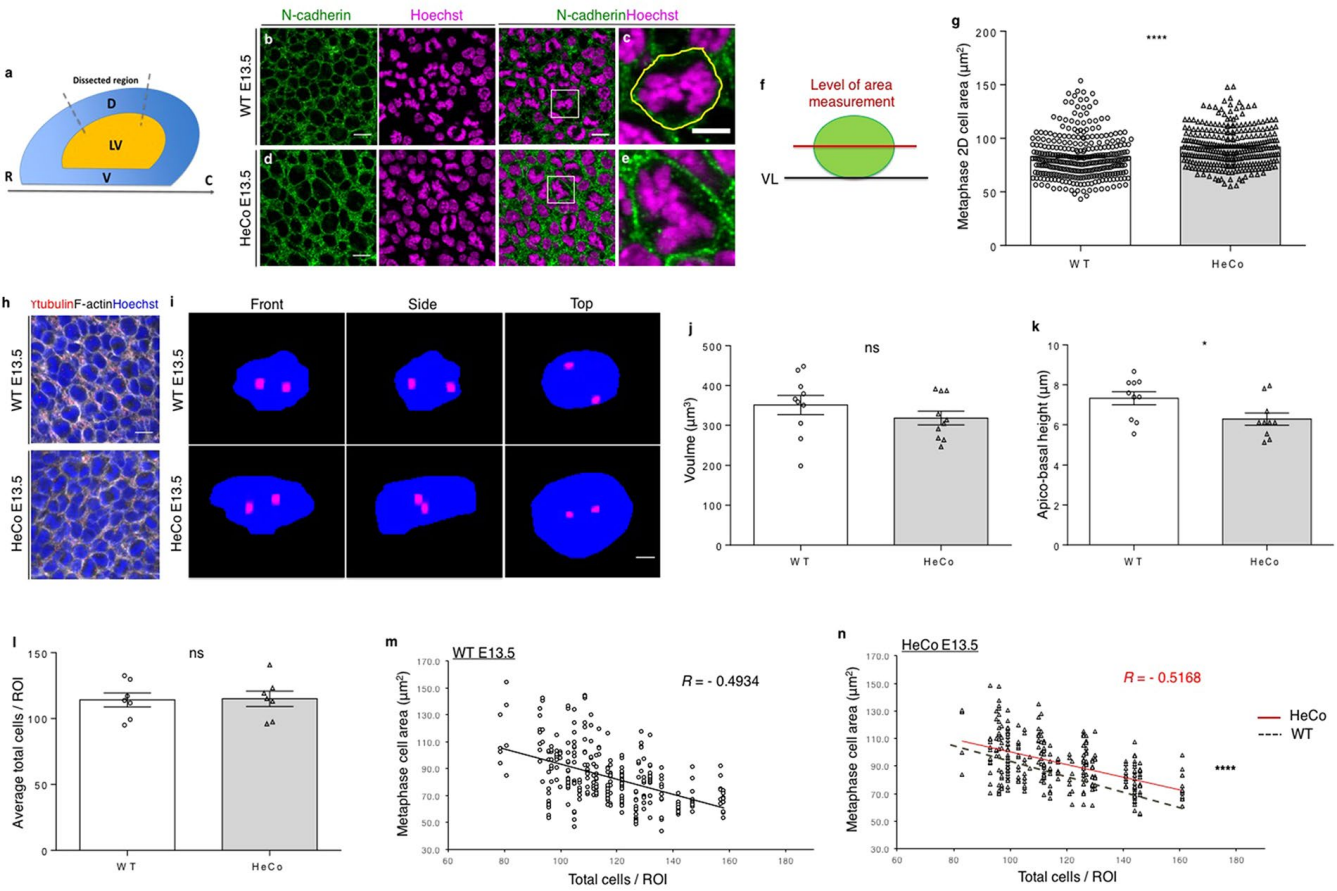
*HeCo* (pro)metaphase somata at the VL have an abnormal shape at E13.5. (**a**) Brain region dissected for *en face* immunohistochemistry (dashed lines). (**b-e**) N-cadherin (green) and Hoechst (magenta) staining for *en face* confocal imaging of WT and *HeCo* VL in E13.5 brains. Higher magnifications (**c,e**) show boxed regions revealing a single metaphase cell. An example of single plane cell area measurement is shown (**c**, yellow line). (**f**) Lateral view of the level (red line) of the *en face* z-stack at which the cell area was measured. Green circle represents the soma of a metaphase cell at the VL (black line). (**g**) Average horizontal area of (pro)metaphase cells is bigger in *HeCo* compared to WT. Single points in the graph represent individual cells. Areas are highly variable (see also **m,n**). (**h**) WT versus *HeCo en face* F-actin (grey) and γ-tubulin (red) staining, combined with Hoechst (blue). (**i**) Representations after 3D segmentation of single (pro)metaphase RGCs. The 3D soma volume is shown in blue from three views (front, lateral, top). Centrosomes (γ-tubulin) are shown in red. (**j**) *HeCo* somata volumes are comparable to WT. (**k**) Somata apico-basal height is reduced in *HeCo* cells. Circles (WT) and triangles (*HeCo*) represent different cells and bars represent the average. (**l**) Cell densities do not differ between WT and *HeCo* E13.5 VLs. Single points in the graph refer to different embryos. Bars represent averages for the two conditions. (**m,n**) Correlations between (pro)metaphase cell areas and total cells/ROI in E13.5 WT (**m**) and *HeCo* (**n**) VLs. Single points in the graph represent single cells. N = 7 embryos from 3 females for each condition for area measurement. WT E13.5 = 37; *HeCo* E13.5 = 34 total ROIs. WT E13.5 = 302; *HeCo* E13.5 = 294 total cell areas. N = 10 cells derived from 3 embryos for each condition for volume segmentation. Unpaired *t*-test (**g,j,k,l**). R, *Pearson*’s correlation coefficients (**m,n**). Linear regression was calculated for comparison of WT and mutant tendencies (**n**). ns, non-significant; *P < 0.05, ****P < 0.0001. LV, lateral ventricle; D, dorsal; V, ventral; R, rostral; C, caudal. Scale bars, 10 µm (**b,d,h**), 5 µm (**c,e**) and 2 µm (**i**).

To further understand the impact of the defect in *HeCo* spindle size, we looked more carefully at the E13.5 VZ. Because the size of the spindle may influence cell size and *vice versa* ^20^, (pro)metaphase cell area was first measured using N-cadherin staining and *en face* imaging (Fig. 5). N-cadherin participates in the assembly of adherens junctions between APs and can thus be used as a marker of cell boundaries^43^. Embryonic dorso-medial brain explants were performed from WT and *HeCo* E13.5 embryos, analyzing the same brain region previously assessed for spindle length (Fig. 5a) and in the area where the heterotopia later develops. Explants were stained for N-cadherin, together with Hoechst, and *en face* confocal imaging^44^ was performed to obtain a clear outline of APs at the VL (Fig. 5b–e). No obvious defects were observed in N-cadherin staining in *HeCo* brains. (Pro)metaphase cells were identified using the whole z-stack based on the characteristic shape of the DNA condensed at the metaphase plate (Fig. 5c,e) and cell area was first measured in a single plane corresponding to the widest cell diameter selected by navigating the apico-basal z-stack (Fig. 5c,e,f). *HeCo* E13.5 single plane cell areas were on average significantly bigger than WT (Fig. 5g). To assess whether the increase in (pro)metaphase cell area reflected an overall increase in the 3D volume of the soma, 10 WT and 10 *HeCo* (pro)metaphase cells were segmented based on F-actin staining, delineating cell boundaries, following *en face* confocal imaging (Fig. 5h,i). Analyses of 3D-segmented mutant (pro)metaphase cells revealed volumes comparable to WT (Fig. 5j). However, the apico-basal height of *HeCo* (pro)metaphase APs was reduced compared to WT (Fig. 5k), leading to a distorted cell shape, with cells being flatter. This data fits the 2D measurements showing increased *en face* single plane areas of *HeCo* (pro)metaphase APs.

To assess the impact on overall tissue morphology of increased horizontal area occupied by (pro)metaphase cell somata, we evaluated cell density in WT and *HeCo* VLs. Indeed, when integrated in a tissue cells adapt to the presence of other cells around them that exert pushing forces proportionate to their density^30^. Due to INM, AP nuclei are only visible at the VL when they are in the G2-M transition, during mitosis, and during M-G1 transition. We thus counted the number of nuclei that were visible in the same *en face* z-stack where the area of (pro)metaphase cells was measured (Fig. 5b,d). The total number of cells was counted in four regions of interest (ROIs) per brain. The average number of nuclei/ROI quantified at E13.5 was found not to differ between WT and *HeCo* brains (Fig. 5l).

To further test cell adaptations within the neuroepithelium, we next checked the horizontal 2D area of (pro)metaphase cells in correlation with the density of nuclei at the VL (Fig. 5m,n). We first found that E13.5 WT (pro)metaphase cell horizontal area was inversely correlated with the total number of nuclei/ROI, with smaller areas found in higher density conditions (Fig. 5m). The same was true for E13.5 *HeCo* APs, however, with increasing total nuclei/ROI, although mutant areas decreased, the elevation of the correlation line became increasingly different from WT (Fig. 5n). Thus, mutant E13.5 (pro)metaphase cells have bigger horizontal areas and whilst both WT and mutant cells appear to reduce their size according to the level of crowding of the tissue, mutant cells occupy more space than WT, this difference becoming more accentuated with increasing cell densities.

## Discussion

This work examines the structural characteristics of the mouse VZ, and especially the most apical region, the VL, where mitosis occurs. We provide new insights into the function of Eml1 in APs and the cellular mechanisms affected by its loss in the *HeCo* developing cortex. *Eml*1 mutant cells show aberrant MT polymerization, centrosomes and primary cilia, mitotic spindle length, as well as cell shape, which together will influence tissue dynamics. WT metaphase cells have relatively round somata and are adaptable, adjusting their diameters depending on cell density. In *Eml*1 mutant conditions, altered cell shape and decreased adaptability may lead to reduced space, eventually causing some progenitors to delaminate. The identification of mislocalized γ-tubulin + puncta, corresponding to centrosomes not associated with dividing cells, supports this hypothesis since these are likely to belong to detaching apical processes^45,46^.

In our previous work at the cellular level, we showed ectopic progenitors in the *HeCo* mouse, but we did not identify any intrinsic defect in post-mitotic neurons^7^. It is indeed possible that other post-mitotic MT factors e.g. Dcx, or other members of the EMAP family, might compensate for Eml1’s role during neuronal development. Indeed, Eml2, Eml4 and Eml5 are all expressed in the mouse developing CP. Heterotopia formation in the *HeCo* model, related to abnormal neuronal migration, is thus likely to be due to extrinsic perturbations, including local cell production and clustering in the IZ, and aberrant RGC guides. Our targeted pull-down experiments reveal a number of Eml1’s potential partners expressed in the VZ, with some having already known functions during the cell cycle. The tight network of partners also highlights roles in MT function and spindle length regulation. Our combined observations suggest that Eml1-dependent MT regulation may be more critical in progenitors than in post-mitotic neurons. Interphase MT growth defects in BLBP+ progenitors, as well as centrosome, primary cilia and spindle length defects in brain slices are in fitting with this. Thus, it is likely that Eml1 plays an MT-dependent role in RGCs at early corticogenesis, not compensated for by other proteins.

To further assess the pertinence of dampened MT dynamics in *HeCo* mutant progenitors *in vitro*, we attempted a rescue experiment. However, Eml1 overexpression in our experimental conditions also decreased MT growth, as shown by similar experiments in N2A cells. Further strategies are hence required to functionally rescue MT growth in mutant progenitors. Due to the difficulty in identifying and live-imaging dividing progenitors in primary cultures, we were not able to track MT growth during mitosis. However, perturbed MT growth in interphase cells, together with the association of Eml1 with mitotic spindles^7^, strongly suggest that MT dynamics could be affected throughout different phases of the cell cycle. This is further suggested by the finding that (pro)metaphase spindle length, strongly influenced by MT dynamics^17^, is abnormal in *HeCo* APs. The growth speed of MTs may indeed impact spindle size^47^. The slower speeds of MT growth we show in this study could have been predicted to lead to shorter MTs and thus shorter mitotic spindles^48–50^. However, recent studies highlight the limitation of classical models of MT growth, especially when explaining the effects of certain MAPs, and suggest that the regulation might be much more complex than initially thought^51^. Indeed, the mitotic spindle is a structure characterized by constant and fast MT rearrangements^33,52^. Thus, due to the complexity of MT dynamics regulation, as well as spindle assembly and function, the slower MT plus-end growth seen *in vitro* in interphase could also be associated with increased (pro)metaphase spindle length in *HeCo* E13.5 APs in the brain.

A large number of MAPs and MT motor proteins are involved in spindle assembly and function^53^, and our combined molecular and cellular data strongly suggest that Eml1 helps regulate these processes. We identified increased spindle lengths during (pro)metaphase stages where the pole-to-pole distance is likely to be near maximum. Despite longer spindles and changed cell shape, no other obvious morphological differences were observed in mutant (pro)metaphase cells. Furthermore, no labeling index or cell cycle exit defects were previously identified in *HeCo* APs in the VZ^7^, suggesting that cell cycle length remains unchanged. The interaction between MT regulators is tightly controlled during the cell cycle, and the removal of one of the players, in this case Eml1, is likely to change the behavior of many other proteins, including Eml1’s partners. Screening WT versus mutant *HeCo* brains by IHC has already revealed Kif22 differences. This MT motor protein plays an established role in mitosis^17,54^ and our new data, although preliminary, strongly suggest an interaction with the Eml1 pathway. Furthermore, we cannot exclude a change in trimerization stoichiometry with other members of the EMAP family, due to the absence of Eml1. Future studies will elucidate the nature of the interaction between Eml1 and candidate partners (as well as those identified in new screens with the full length protein) and clarify how Eml1 loss and/or mutation, including missense mutations identified in patients, impacts the function of these molecules.

**Figure 6:**
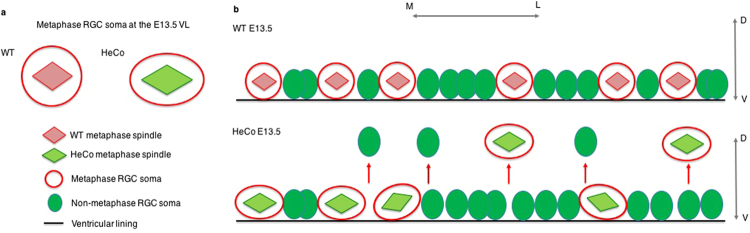
Schematic representation of spindle length and cell shape, and delamination hypothesis for *HeCo* APs. (**a**) Lateral view of spindle length and cell shape differences in WT and *HeCo* metaphase cells dividing at the VL. *HeCo* cells have a longer spindle and a flatter soma. (**b**) Delamination hypothesis for *HeCo* progenitors based on the interplay between spindle length, cell density and metaphase cell shape. At E13.5 in the WT a number of metaphase cells are present at the VL. At E13.5 in *HeCo*, spindles are longer and metaphase cells flatter, which reflects in an increase in the horizontal (with respect to the VL) space they occupy. This may cause increased mechanical stress in the VZ and especially at the VL and encourage delamination (red arrows), with some RGCs pushed outside the VZ during early-corticogenesis. For simplicity the VZ is not represented as pseudo-stratified but only cell somata at the VL are shown, without RGC apical and basal processes. M, medial; L, lateral; D, dorsal; V, ventral.

*HeCo* E13.5 spindles in the VZ show increased pole-to-pole distance during (pro)metaphase, and we propose that this may be why these mutant cells are abnormally shaped, elongated in the medio-lateral axis but shorter in the apico-basal axis, and hence flatter than WT cells, at a stage of corticogenesis in which spindles are mostly oriented horizontal to the VL and RGCs frequently undergo self-renewal (Fig. 6a). Because of the potential role of Eml1 in the regulation of MT dynamics and its probable interaction with spindle length regulators, it seems reasonable to think that the perturbation in spindle size may change cell shape, instead of *vice versa*. Indeed, a longer spindle in *HeCo* progenitors may lead astral MTs to relax the cell poles and produce cell elongation along the spindle axis^55^. This is further suggested by the fact that the average difference in horizontal area of (pro)metaphase cells between WT and mutant increases in regions of the VZ where cellular nuclei are denser (Fig. 5l–n). This suggests that intrinsic properties of mutant cells may hamper the adaptation of somata to cell density in brain tissue.

Assessing (pro)metaphase spindle length at an earlier time-point might reinforce the hypothesis that (pro)metaphase cell defects contribute to ectopic progenitor localization. Indeed, Eml1 transcripts are detectable in the VZ at E12.5 (Supplementary Fig. S1a) and it is thus possible that spindle length as well as other RGC features described here may begin to show alterations already at these earlier stages of corticogenesis. In addition, providing spindle length and cell shape data for later stages of development in both WT and mutant brains would also be interesting to shed further light on progenitor behavior during corticogenesis. The cortical VZ is known to change over time, including transitions from symmetrical to asymmetrical division^1^, and testing tissue dynamics in this way could contribute to our overall comprehension of cortical development.

We have focused here on the mutant VZ but an important area also concerns abnormal cells in the IZ. We show in *HeCo* early-mid corticogenesis that spindle lengths of dividing IZ mutant cells appear comparable to APs dividing at the VL, which also favors the intrinsic nature of the spindle length phenotype. Ectopic progenitors are likely to provide a local source of daughter cells including post-mitotic neurons. It remains to be seen if some perturbed protein partners also contribute to progenitor cell/neuron clustering in the IZ which may be important for heterotopia formation.

Concerning the VZ phenotypes, there may be additional roles for Eml1 in other MT-related functions that influence cell shape, which in turn may explain the increase in spindle length. This prompted us to verify primary cilia in *HeCo* brains with the Arl13b ciliary marker. The reduced staining indicates less numerous and/or defective primary cilia in the VL. Together with centrosome basal displacement, the reduced primary cilia in the mutant VZ may indicate AP apical process detachment. The reduction of primary cilia suggests that these organelles may be excised and secreted into the ventricle, while the apical process of the delaminating AP retracts, carrying along with it the centrosome. Indeed, the budding and shedding of primary cilium-derived particles from APs into the ventricles has been previously described^56^. Apical process retraction followed by shedding of primary cilium-related particles was also described in the chick neuroepithelium for newly produced neurons leaving the VL^57^. In *HeCo* mice, reduced primary cilia in the VL may be a consequence of cell delamination, however in this case, it is mutant Pax6 + progenitors that escape from the VZ. Future studies are required to further understand this phenotype and it will also be important to investigate potential anomalies in signaling pathways, such as mTOR, Wnt and Shh, in which the primary cilium appears to be a critical organelle^58^. It is premature to conclude in the *HeCo* model that primary cilia defects are responsible for the detected cell shape and spindle length anomalies, nevertheless this warrants further analyses. Alternatively, as well as intrinsic spindle perturbations, cell membrane properties could also influence the shape of the cell and its response to mechanical stress, allowing e.g. responsiveness of the membrane on the basal side but less adaptability horizontally. It is interesting to note that in *C. elegans* it has been suggested that the Emap Elp, plays a role in mechano-transduction^59^. Considering the role of Eml1 in each of these functions should help clarify the mechanisms by which mutant cells show abnormal shape.

We reveal changes in spindle length and cell shape at early-mid corticogenesis that might impact RGC position and cell fate. We hypothesize that for mechanical reasons and tissue constraints, some mutant cells may begin to be pushed away from the VZ, contributing to the presence of ectopic progenitors in the IZ and CP (Fig. 6). Indeed, delamination is a known response to increased cell densities in other epithelial tissues^60,61^, and has also been identified in one other study in the cortical VZ^28^. Whether delamination is initiated during mitosis by impacting spindle lengths and orientations, or in interphase, via cell signaling leading directly to apical endfoot detachment, is currently not clear. Live-imaging experiments during the cell cycle with centrosome and chromatin markers may shed further light on primary events, as well as possibly identifying further mitotic defects. It is important to identify these cellular abnormalities since they initiate a series of detrimental steps affecting corticogenesis, with the severe consequences of heterotopia.

## Materials and Methods

### Animals

Research was conducted according to national and international guidelines (EC directive 86/609, French MESR 00984.02) with protocols followed and approved by local ethical committees (Charles Darwin committee (Paris, France) and Office Vétérinaire Cantonal (Lausanne, Switzerland). *HeCo* mutant mice arose spontaneously in a colony of NOR-CD1 outbred stock, and selective inbreeding including crossing of living relatives and backcrossing were used to increase the occurrence of the phenotype in offspring as described in ref.^6^. WT and *HeCo* mice derived by separate but regularly crossed colonies were used for developmental analyses and primary neuronal cultures. The mode of inheritance of the phenotype is autosomal recessive. Normal, full-length transcripts of *Eml1* are absent in *HeCo* brains due to the insertion of an early retrotransposon (ETn) in the last intron of the gene^7^, and are replaced by trace levels of shortened and chimeric Eml1-ETn transcripts. Timed-pregnant Swiss mice used for embryonic cortex lysate preparation were provided by Janvier Labs (http://www.janvier-labs.com/home.html). For staging of embryos, the day of vaginal plug was considered E0.5. Mice were housed with a standard 12 h light/dark schedule (lights on at 07:00 a.m.).

### MT plus-end tracking – video-microscopy

Primary cultures of cortical progenitors were prepared from E12.5 embryos derived from timed-pregnant NORCD1 WT and *HeCo* mice. Cortices from both hemispheres were dissected in ice-cold Leibovitz-15 (L-15, Gibco BRL) medium. After removal of the meninges cortices were washed in 4 °C dissection-dissociation medium (HEPES 20 mM, HBSS 1X, Life Technologies). Cells were mechanically dissociated in DMEM Glucose (Life Technologies) supplemented with 10% Fetal Calf Serum (FCS, Thermo Scientific) before electroporation with BLBP-IRES-EGFP and EB3-mCherry (3 μg total DNA) using an Amaxa mouse Nucleofector kit (Lonza). Rescue experiments in *HeCo* progenitors were performed with pBLBP–Eml1–IRES–GFP^7^. Neuro-2A cells were transfected with either EGFP alone (pEGFP C3, Clontech) or EGFP-Eml1, in combination with EB3-mCherry (see Supplementary Information for Neuro-2A cell culture). Cells were seeded in 35 mm diameter glass bottom Ibidi dishes suitable for video-microscopy previously coated with poly-L-lysine and laminin (Sigma-Aldrich). Progenitors were cultured and maintained in B27/N2 medium (Gibco BRL), which is a mixture (1:4) of Neurobasal/B27 medium without vitamin A and DDM medium (DMEM/F12 with Glutamax, supplemented with N2, 0.1 mM nonessential amino acids, 1 mM sodium pyruvate, 500 μg/ml BSA, 0.1 mM 2-mercaptoethanol and Primocin 100 U/ml, Lonza). EGFP+ cells were filmed at 2 DIV. Video-microscopy was performed using a Spinning Disk rapid inverted confocal (Leica DMI4000) equipped with a temperature-maintaining chamber and an intensified camera, and piloted by Metamorph. A 63X objective and 591-laser were used to film EB3-labeled growing MT plus-ends during 2 min with a time-interval of 1 sec (3 z-stacks of 300 ms exposure per time-frame). Tracking was performed on stack images using the Manual Tracking plugin of the ImageJ software. Only EB3+ comets recognizable during at least 5 consecutive time points were considered for analysis.

### Immunohistochemistry on coronal sections

Mouse embryonic brains were fixed by immersion overnight (O/N) at 4 °C in 4% w/v paraformaldehyde (PFA) in 0.1 M phosphate buffer, pH 7.4. Brains were cut in 70 µm thick coronal sections using a vibrating blade microtome (Leica VT1000 S). Blocking was performed for 1 hour at RT with blocking solution (PBS 1X with 10% Goat Serum and 0.5% Triton X-100) before incubation O/N at 4 °C with the following primary antibodies: mouse γ-tubulin (GTU-88, T6557, Sigma-Aldrich, 1:500), rabbit Arl13b (17711-1-AP, Proteintech, 1:500), rabbit Kif22 (13403-1-AP, Proteintech, 1:150), rabbit Kif18A (1925-1-AP, Proteintech, 1:150), rabbit Dync1h1 (R-325, sc-9115, Santa Cruz Biotechnology, 1:200). After extensive washes, sections were incubated with secondary anti-mouse Alexa 488 or anti-rabbit Alexa 568 (Life Technologies, 1:800-1:1000). Antigen retrieval was performed before the blocking step for Kif18A and Dync1h1 antibodies. For this, sections were incubated in sodium citrate 10 m: mM pH 6 at 95 °C for 20 minutes and cooled down before blocking. Sections were subsequently incubated with Hoechst 1:1000-1:5000 and mounted with Fluoromount G (Southern Biotechnology). Fluorescently stained sections were imaged with confocal microscopes (Olympus FV10i and TSC Leica SP5-II) equipped with 10x phase contrast objective/NA 0.4 and 60x phase contrast oil-immersion objective/NA 1.35, and 10x, 40x oil Plan-Neofluor, 63x, 100x oil Plan-Apochromat objectives. Fluorophore excitation and scanning were performed with argon lasers at 488 nm (blue excitation for GFP, Alexa 488) and 568 (red excitation for Alexa 568), and with a diode laser at 405 nm (for Hoechst staining). Confocal images were acquired with a 0.17 µm or 0.3 µm z-stack depth. Images were analyzed using Image J (Fiji) to obtain the whole *z*-stack data set and for γ-tubulin and Arl13b quantification. At least two ROIs of 120 × 35 µm were quantified per embryo. The Imaris software was used for single-cell 3D reconstruction and spindle length measurement. Single (pro)metaphase cells were recognized from DNA shape and centrosome position and cropped from whole-section images to isolate them. Z-stacks of cropped single cells were reconstructed in 3D by applying voxel depth, and pole-to-pole distance was measured.

### Pull-down

E13.5 timed-pregnant Swiss mice (Janvier Labs, France) were sacrificed by cervical dislocation. Embryos were dissected and both brain hemispheres collected in L-15 medium, and explants immediately frozen in liquid nitrogen and ground into a powder. This was re-suspended in 10 μl/mg lysis buffer (Tris HCl 50 mM, NaCl 150 mM, EDTA 1 mM pH 8) supplemented with 1% NP-40 and protease inhibitors 1X (Protease Inhibitor Cocktail Tablets EDTA-Free, Sigma-Aldrich). The lysate was homogenized by rotation during 45 min at 4 °C, then centrifuged for pre-clearing 30 min at 15000 rcf and 4 °C. Cortex extracts (15 μg tissue) were incubated overnight with pre-washed Glutathione-Agarose resin (Sigma- Aldrich) previously coupled with either purified GST-EML1 N-ter^36^ or GST as control (4 μg total purified protein). Extracts were centrifuged (200 rcf, 1 min) to pellet the resin, supernatants were collected as un-bound fractions, and resins (bound fractions) were extensively washed with lysis buffer to remove non-specific interactions. Resins were re-suspended in freshly-made Laemmli buffer, heated 10 min at 95 °C and centrifuged 2 min at maximum speed to dissociate the complexes from the resin and denature the proteins.

The EML1 N-ter construct, is predicted to bind MTs and not soluble tubulin^36^. Indirect protein partners due to MT-mediated interactions were not favored because of the sample preparation conditions (4 °C cold treatment causing depolymerization). The unlikely occurrence of soluble tubulin-mediated interactions was further confirmed by detection in Western blots of pull-down samples identifying α-tubulin only in the unbound fractions (Supplementary Fig. S3).

### Mass Spectrometry

Two independent pull-down purifications (GST and GST-EML) were simultaneously separated by SDS-PAGE and stained with colloidal blue (LabSafe Gel Blue GBiosciences). Seven gel slices were excised for each purification. After washing, proteins were reduced with 10 mM DTT prior to alkylation with 55 mM iodoacetamide. After washing and shrinking of the gel pieces with 100% acetonitrile, in-gel digestion was performed using trypsin (Gold, Promega) overnight in 25 mM ammonium bicarbonate at 30 °C. Peptides extracted from each band were analyzed by nano Liquid Chromatography (LC)-MS/MS using an Ultimate 3000 system (Dionex S.A.) coupled to an LTQ-Orbitrap XL mass spectrometer (Thermo Scientific). Data-dependent acquisition was performed in the positive ion mode. Survey MS scans were acquired in the 475–1200 m/z range for each sample, with the resolution set to a value of 60 000. Each scan was recalibrated in real time by co-injecting an internal standard from ambient air into the C-trap (‘lock mass option’). The 5 most intense ions per survey scan were selected for CID fragmentation and the resulting fragments were analyzed in the linear trap (LTQ). Target ions already selected for MS/MS were dynamically excluded for 180 s. Data were acquired using the Xcalibur software and the resulting spectra analyzed via the MascotTM Software (Thermo Scientific). All peptide/protein identification data were further processed using the Institut Curie developed software myProMS (http://myproms.curie.fr/)^70–72^, version 3.0. The mass spectrometry proteomics data were deposited to the ProteomeXchange Consortium via the PRIDE^73^ partner repository with the dataset identifier PXD006837. Protein lists were analyzed using the DAVID Functional Annotation Tool^38^ (https://david.ncifcrf.gov/home.jsp) for Gene Ontology generation, and STRING Functional Protein Association Network^39^ (string-db.org) to reveal interactions between proteins. See also Supplementary Information for more details on MS data analysis.

### *En face* immunohistochemistry

Following the protocol adapted from ref.^44^, mouse embryonic brains were fixed in 4% w/v PFA (Sigma-Aldrich, France). Cortical explants were dissected and incubated 15 min at RT in PBST 1% (PBS 1X containing 1% Triton X-100 v/v and 0.02% sodium azide). Explants were then incubated 2 h at RT in blocking solution (PBS 1X, 0.3% Triton X-100 v/v, 0.02% sodium azide, 3% w/v Bovine Serum Albumin). Primary antibody mouse monoclonal anti-N-cadherin (C70320, Transduction Laboratories, 1:2000) or γ- tubulin (GTU-88, T6557, Sigma-Aldrich, 1:500) were applied O/N at RT. After extensive washing in blocking solution explants were incubated O/N at RT with secondary antibody anti-mouse Alexa 488 (1:800, Thermo Fisher Scientific) together with Hoechst (1:1000-5000, Thermo Fisher Scientific). Washes in blocking solution and PBS 1X, were performed before mounting the explants with Fluoromount G positioned as flat as possible with the ventricular surface up to obtain an *en face* view of the ventricular side of the cortex. For F-actin immunofluorescence, Alexa Fluor 633 Phalloidin (1:100, Life Technologies) was incubated in PBST 1% O/N at RT. Extensive washing was performed in PBST 1% and PBS 1X before mounting the explants. Fluorescently stained sections were imaged as previously described. Confocal images were acquired with a 0.2 µm z-stack depth for a total depth of 9–10 µm (Olympus FV10i microscope and TCS Leica SP5-II). At least two randomly-chosen ROIs were imaged for each hemisphere. Images were analyzed using Image J (Fiji) to obtain the whole *z*-stack data set and the cell counter and measuring plugins for quantification. Cell counting and single metaphase cell area measurements were performed in the same ROIs (100 × 100 µm) and on the first layer of cells starting from the VL. Areas were measured on the z-stack corresponding to the largest diameter of metaphase cells. For cell volume and height measurements, individual (pro)metaphase cells were recognized from DNA shape and centrosome position, and cropped for manual segmentation. 3D-reconstruction of cropped single cells was done with the Imaris software by tracing manually cells’ contours (from stained F-actin), and interpolating all contours with a surface in 3D.

### Plasmids

The pGEX-EML1-174 and GST control constructs are as described in ref.^36^. The BLBP-IRES-GFP construct was obtained from the N. Heintz laboratory (Rockefeller University, New York) and modified to include Eml1^7^. The EB3-mCherry plasmid was a kind gift from A. Andrieux (Grenoble Institute of Neurosciences, Grenoble, France).

### Statistical analysis

Statistical analyses were performed using StatView, BiostaTGV (marne.u707.jussieu.fr/biostatgv/) and GraphPad Prism. Normal distribution of the data was verified before applying statistical tests. The unpaired *t*-test was applied to compare WT and *HeCo* EB3 tracking, γ-tubulin and Arl13b + puncta, spindle length, metaphase cell area, volume and height. *Pearson’s* correlation coefficients and respective *P*-values were calculated. Linear regression tests were applied for comparison of correlations. No data points were excluded. All data were processed in a blind manner. No statistical methods were used to predetermine sample sizes, but our sample sizes are similar to those generally employed in the field^7,10,58^.

### Data availability

The datasets generated during and/or analyzed during the current study are available from the corresponding author on reasonable request.

## Electronic supplementary material

Supplementary Information

Supplementary Video S1

Supplementary Video S2

Supplementary Table S1

Supplementary Table S2
