# Supplementary material for: Eml1 loss impairs apical progenitor spindle length and soma shape in the developing cerebral cortex
Source: Sci Rep. 2017 Dec 11;7:17308. doi: 10.1038/s41598-017-15253-4 (PMC5725533; doi:10.1038/s41598-017-15253-4)
Supplement: Supplementary file 1 — Supplementary Information [file 41598_2017_15253_MOESM1_ESM.pdf]

Eml1 loss impairs apical progenitor spindle length and soma shape in the developing cerebral cortex

Sara Bizzotto[1-3,#,§], Ana Uzquiano[1-3,#], Florent Dingli[4]; Dmitry Ershov[5], Anne Houllier[1-3], Guillaume Arras[4]; Mark Richards[6], Damarys Loew[4]; Nicolas Minc[5], Alexandre Croquelois[7,8], Anne Houdusse[9], Fiona Francis[1-3,*]

[1] INSERM UMR-S 839, 17 rue du Fer à Moulin, Paris 75005, France.

[2] Sorbonne Universités, Université Pierre et Marie Curie, 4 Place Jussieu, Paris 75005, France.

[3] Institut du Fer à Moulin, 17 rue du Fer à Moulin, Paris 75005, France.

[4] Institut Curie, PSL Research University, Centre de Recherche, Laboratoire de Spectrométrie de Masse Protéomique, 26 rue d'Ulm, 75248 Cedex 05 Paris, France.

[5] Institut Jacques Monod, UMR7592 CNRS, Paris, France.

[6] Department of Biochemistry, University of Leicester, Henry Wellcome Building, Lancaster Road, Leicester, LE1 9HN, U.K.

[7] Department of Clinical Neuroscience, Centre Hospitalier Universitaire Vaudois and University of Lausanne, 21 rue du Bugnon, 1011 Lausanne, Switzerland.

[8] Department of Fundamental Neurosciences, University of Lausanne, 1005 Lausanne, Switzerland.

[9] Structural Motility, Institut Curie, Centre de Recherche; CNRS, UMR144, 26 rue d'Ulm, Cedex 05, Paris 75248, France.

[§] Current affiliation: Division of Genetics and Genomics, Manton Center for Orphan Disease, and Howard Hughes Medical Institute, Boston Children's Hospital, Boston, MA, USA. Departments of Pediatrics and Neurology, Harvard Medical School, Boston, MA, USA. Broad Institute of MIT and Harvard, Cambridge, MA, USA.

[#] These authors contributed equally to this work.

**Corresponding author*: Fiona Francis, Institut du Fer à Moulin, 17 rue du Fer à Moulin, 75005 Paris France. Tel: +33145876145 Email: fiona.francis@inserm.fr

Supplementary information

Supplementary Materials and Methods

***In situ* hybridization.** Mouse digoxigenin (DIG)-labeled riboprobes were generated by *in vitro* transcription of a fragment amplified from the *Eml1* 3′ UTR region (chr12 nt 109776616 to 109777469, Genome Browser, http://genome.ucsc.edu/) and subcloned in pBluescript II KS vector (7). Frozen 20 µm thick cryostat sections were hybridized at 65°C overnight with the DIG-labeled probes diluted 1:100 in hybridization buffer (50% deionized formamide, 10% dextran sulphate, 1 mg/ml Yeast RNA, 1x Denhardt's solution). The next day, sections were sequentially washed twice (45 minutes) in 1X saline sodium citrate (SSC), 50% formamide and Tween 0.1% at 65°C. Sections were washed extensively with PBS Tween 20 (PBST) 0.1% followed by PBS 1X. For immunological detection of DIG-labeled hybrids, sections were first blocked (10% sheep serum, 0.1% Tween in PBS 1X) and then incubated overnight at 4°C in the same solution containing sheep anti-DIG-alkaline phosphatase-conjugated Fab fragments (Roche Diagnostics) diluted 1:2000. The following day, sections were washed 3 x 15 minutes in PBST 0.1% and 30 min in NTMT buffer (100 mm NaCl, 100 mm Tris-HCl, pH 9.5, 50 mm MgCl2, 0.1% Tween 20). The alkaline phosphatase chromogen reaction was performed in NTMT buffer containing 100 mg/ml nitroblue tetrazolium (Roche Diagnostics) and 50 mg/ml 5-bromo-4-chloro-3-indolyl phosphate (Roche) at room temperature for 2-4 hours and stopped with PBS 1X. Sections were mounted with Mowiol. Images were acquired with an EVOS Digital Microscope.

N2A cell transfection and culture. Cells were cultured in 10% fetal bovine serum (FBS)-supplemented Dulbecco Modified Eagle Medium (DMEM) (Sigma-Aldrich). N2A cells were co-transfected with either pEGFP-C3 control vector (Clontech) or pEGFP-Eml1, and EB3-mCherry (10 µg total DNA) using the Neon Transfection System (Thermo Fisher Scientific), and seeded in 35 mm diameter glass bottom Ibidi dishes suitable for video-microscopy. Video-microscopy was performed as for neural progenitors at 2 DIV.

MS data processing and statistical analysis. Data were acquired using the Xcalibur software and the resulting spectra were analyzed via the MascotTM Software created with Proteome Discoverer (Thermo Scientific) using the *Mus musculus* database containing 16587 protein sequences. Carbamidomethylation of cysteines, oxidation of methionine and protein N-terminal acetylation were set as variable modifications for searches. Specificity of trypsin digestion was set and two missed cleavage sites were allowed. The mass tolerances in MS and MS/MS were

set to 2 ppm and 0.5 Da, respectively. All peptide/protein identification data were further processed using the Institut Curie developed software myProMS (http://myproms.curie.fr/) (62) version 3.0. The false discovery rate (FDR) for peptide identification was fixed to less than 1% at the peptide level for the whole study, and the QVALITY algorithm (63) was used. For label-free quantification, peptide XICs (Extracted Ion Chromatograms) were computed with MassChroQ v1.2.1 (73) using OBI-Warp alignment algorithm. Protein ratios were computed as the geometrical mean of related peptides. To estimate ratio significance, a two-tailed *t*-test was performed with a Benjamini–Hochberg FDR control threshold set to 0.05. Data were analyzed using the DAVID Functional Annotation Tool (38) (https://david.ncifcrf.gov/home.jsp) for Gene Ontology generation, and STRING Functional Protein Association Network (string-db.org) (39) to reveal interactions between proteins.

Supplementary Video legends

Supplementary Video S1. **Example of WT EB3-mCherry live-imaging.** E12.5 mouse WT cortices were dissected and neural progenitors were dissociated and co-transfected with plasmids expressing EB3-mCherry and the RGC marker BLBP-EGFP. Cells were cultured at 37°C and 5% CO_2 two days before imaging. EGFP-positive cells were imaged for EB3-mCherry (red) using time-lapse confocal microscopy and Metamorph software during 2 min with frame acquisitions every 1 sec. Three confocal planes were acquired (300 ms exposure) and z-stack projections were made for each frame. Display rate: 10 frames/second. Related video still is shown in Figure 1a.

Supplementary Video S2. **EB3 tracking.** Higher magnification of one cell shown in Supplementary Video 1 and Figure 1a showing EB3 tracks. Each colored track represents a single EB3-positive MT. EB3-mCherry is shown in black on a white background. Tracks were generated manually using the Manual Track ImageJ plugin on stack images corresponding to 1 frame generated by max projection of three confocal planes. Display rate: 10 frames/second. Related video still is shown in Figure 1a (higher magnification).

Supplementary Table legends

Supplementary Table S1. **List of the 1059 proteins uniquely associated with GST-EML1 N-ter samples based on XIC quantification.** Gene ontologies based on the three categories: biological process (BP), cellular component (CC), and molecular function (MF) were generated using the DAVID functional annotation tool.

Supplementary Table S2. **List of the 176 proteins that passed filtering of the 1059 proteins uniquely associated with GST-EML1 N-ter**. Gene ontologies based on the three categories biological process (BP), cellular component (CC), and molecular function (MF) were generated using the DAVID functional annotation tool. A list of 10 kinesins is also reported.

Supplementary Figures

Supplementary Figure S1

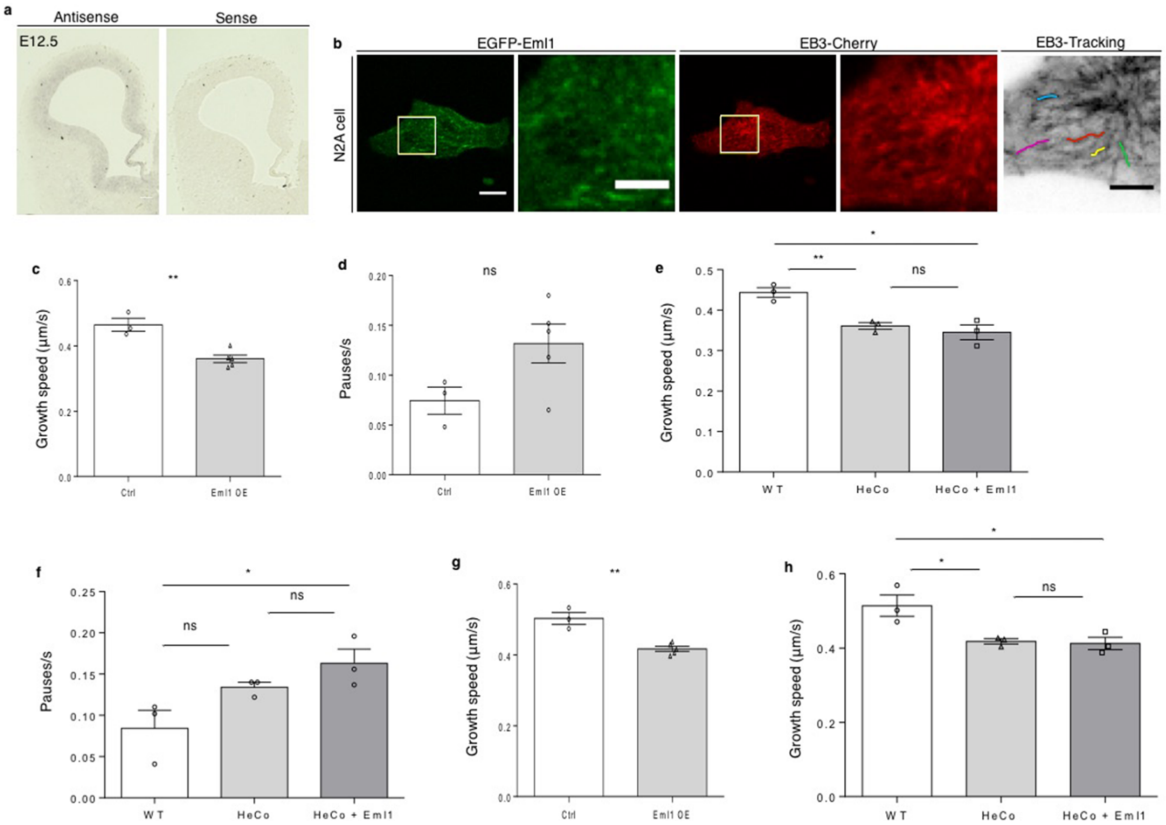

Supplementary Figure S1. **Eml1 overexpression reduces MT growth in N2A cells and does not rescue MT dynamics in *HeCo* progenitors. (a)** Eml1 transcripts are detected in the cortical VZ of WT E12.5 embryos. (**b**) N2A cells co-transfected with either EGFP (Ctrl) or EGFP-Eml1 and EB3-mCherry. Fluorescent EB3 was filmed with a confocal microscope equipped with a spinning disk and EB3-positive plus-ends tracked using Image J. Colored lines in the far right image show a representation of tracking. Higher magnifications of the boxed areas are shown. (**c**) EGFP-Eml1 overexpression (Eml1 OE) reduces MT growth rate. (**d**) Pause frequency during MT growth does not change significantly upon Eml1 OE although there is a tendency for increased pauses. Ctrl, N = 3; Eml1 OE, N = 4 independent experiments. Circles and triangles in the graphs represent different experiments. A total of 19 cells were analyzed for both Ctrl and Eml1 OE. Ctrl, 114; Eml1 OE, 102 total single EB3-positive MTs tracked. (**e,f**) EB3-MT growth in neural progenitors from dissociated cultures prepared from E12.5 embryonic cortices. First two bars shown for comparison represent the same data as shown in Figure 1b. WT and *HeCo* progenitors were transfected with either BLBP-IRES-EGFP or BLBP-Eml1-IRES-EGFP and EB3-mCherry. EB3 comet tracking was performed and quantified in EGFP+ cells. MT growth speed is still reduced upon transfection of Eml1 in *HeCo* progenitors compared to WT and does not change compared to *HeCo* (**e**). Eml1 overexpression in *HeCo* progenitors significantly increases the MT pause frequency compared to WT but not compared to *HeCo* (**f**). (**g,h**) Growth rate comparisons without taking into account pauses give results comparable to when pauses are included in the calculation, for both N2A cells (**g**) and neural progenitors (**h**). N= 3 independent experiments for each condition. *HeCo* +Eml1, 17 cells analyzed for a total of 156 single MTs tracks. Unpaired *t*-test, ns, non significant; * $P < 0.05$; ** $P < 0.01$. Scale bars 100 µm (**a**), 10 µm (**b**) and 5 µm (high magnifications).

Supplementary Figure S2

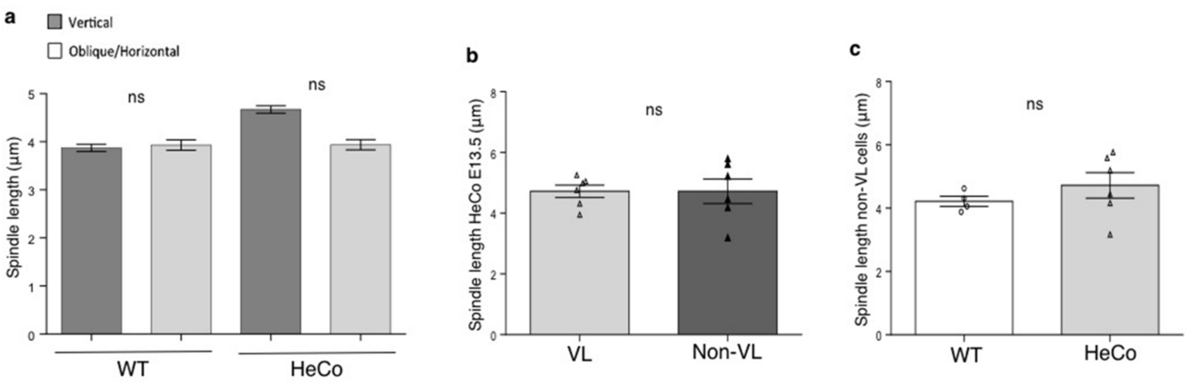

Supplementary Figure S2. **Spindle length does not change between vertical and oblique/horizontal spindle orientations and *HeCo* VL and non-VL progenitors.** (**a**) Spindles were classified from 3D-reconstructed single (pro)metaphase cells at the VL as having vertical or oblique/horizontal cleavage planes (DNA plate orientation with respect to the VL) and average lengths were compared for the two categories for each genotype and developmental stage. No significant differences were observed. (**b,c**) Spindle length was measured in WT and *HeCo* brains in cells dividing within the VZ but far from the VL as well as in cells dividing outside the VZ (WT = 19, *HeCo* = 31 total cells). No significant difference was found between VL and non-VL cells in *HeCo* brains. Spindle lengths are more variable in non-VL *HeCo* dividing cells. This variability could be due to an increased sensitivity of the latter to external forces, and/or to increased spindle length oscillations in (pro)metaphase when cells are located in this region. Variability may also be due to the fact that other types of basally located cells may be included in the analysis, as well as ectopic apical progenitors. (**b**) Spindle length of VL cells is not significantly different between WT and *HeCo*, however, there is a tendency for longer spindles in *HeCo* basal (pro)metaphase cells (**c**). Unpaired *t*-test. ns, $P > 0.05$.

Supplementary Figure S3

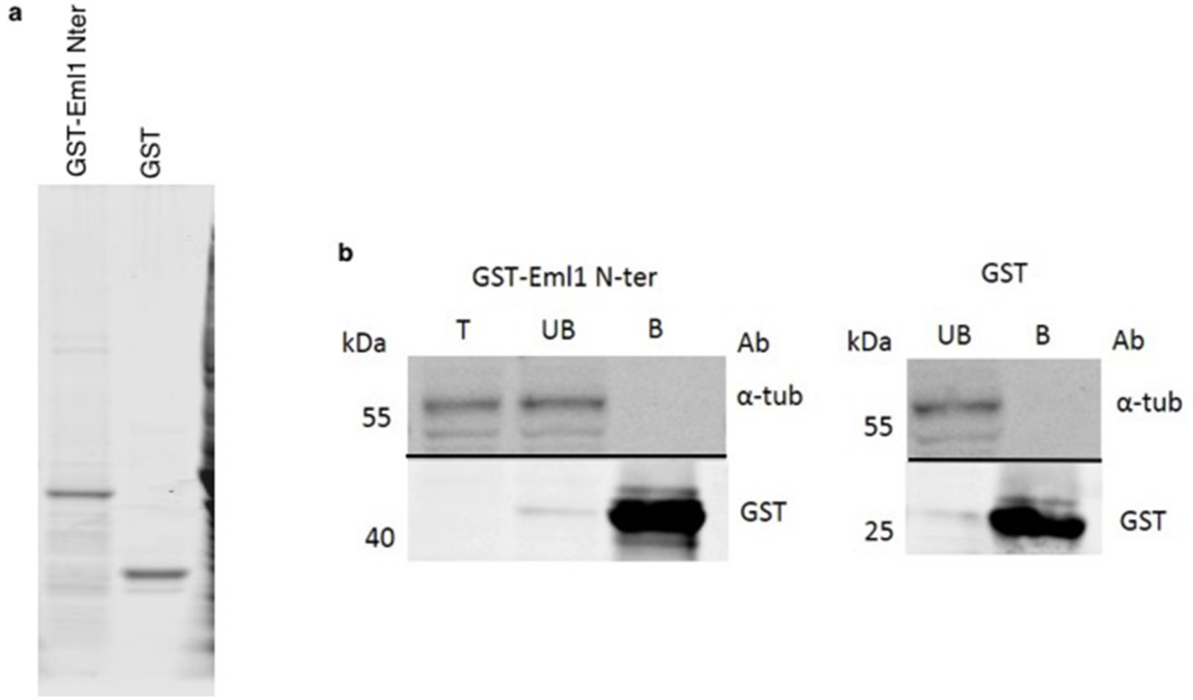

Supplementary Figure S3. **Controls for GST-EML1 Nter pull-down.** (**a**) Control gel with pull-down samples for GST-EML1 N-ter and GST control stained with Colloidal Blue. (**b**) Western Blot showing no binding of alpha-tubulin to GST-EML1 N-ter after pull-down. Black lines separate blots which were not run together, see Supplementary Figure S5 for full blots. Antibodies: mouse anti-alpha-tubulin 1:10000 (DM1A clone, Sigma-Aldrich T9026), rabbit anti-GST 1:1000 (Sigma-Aldrich, G7781) T, total fraction; UB, un-bound fraction; B, bound fraction.

Supplementary Figure S4

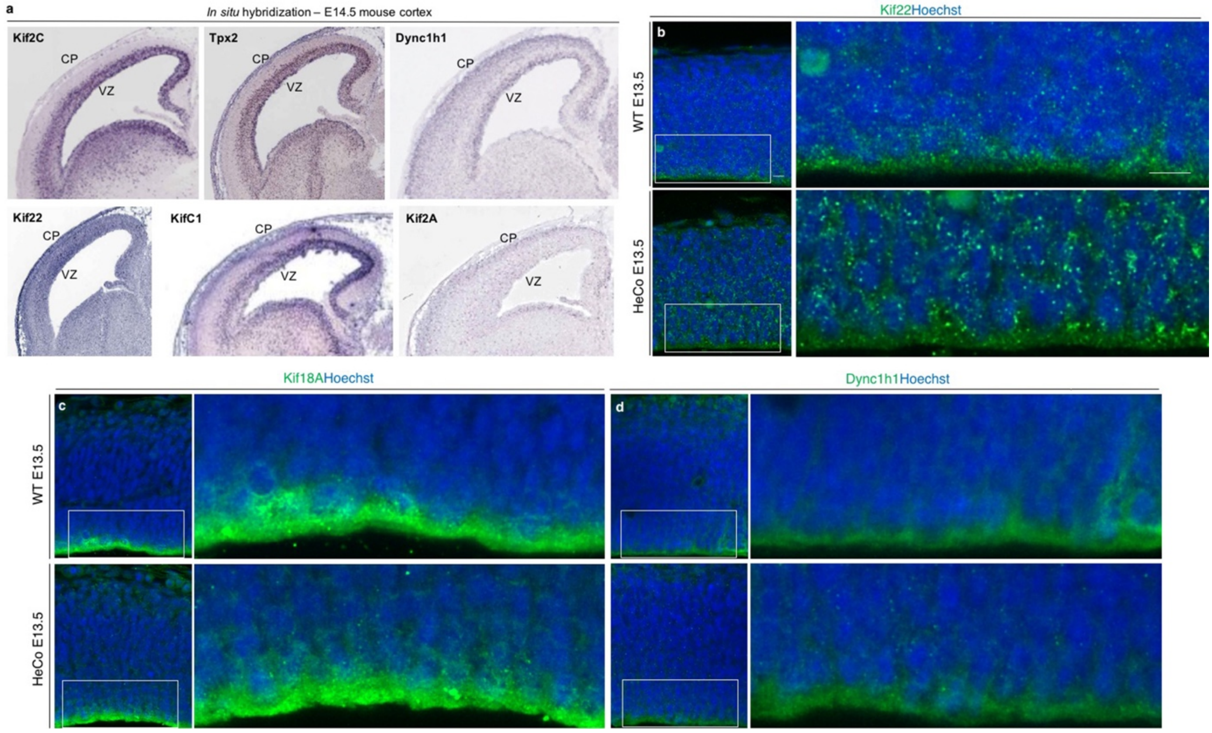

Supplementary Figure S4. **MS proteins involved in spindle length regulation are expressed in the mouse VZ, and the microtubule motor protein Kif22 shows a different pattern of expression in *HeCo* developing cortex.** (**a**) *In situ hybridization* images obtained from publicly available resources (http://www.genepaint.org) showing *Kif*C1, *Kif*2A, *Kif*22 and *Dync*1h1 expression in the VZ and CP of the mouse developing cortex at E14.5, and expression of *Kif*2C and *Tpx*2 in the VZ only. (**b**) IHC for Kif22 in WT and *HeCo* E13.5 cortices showing altered expression in *HeCo* VZ. White boxes delineate the higher magnification images. (**c,d**) Kif18A and Dync1h1 IHC show similar levels of the proteins and similar expression patterns in WT versus *HeCo* E13.5 cortices. Scale bars 10 µm.

Supplementary Figure S5

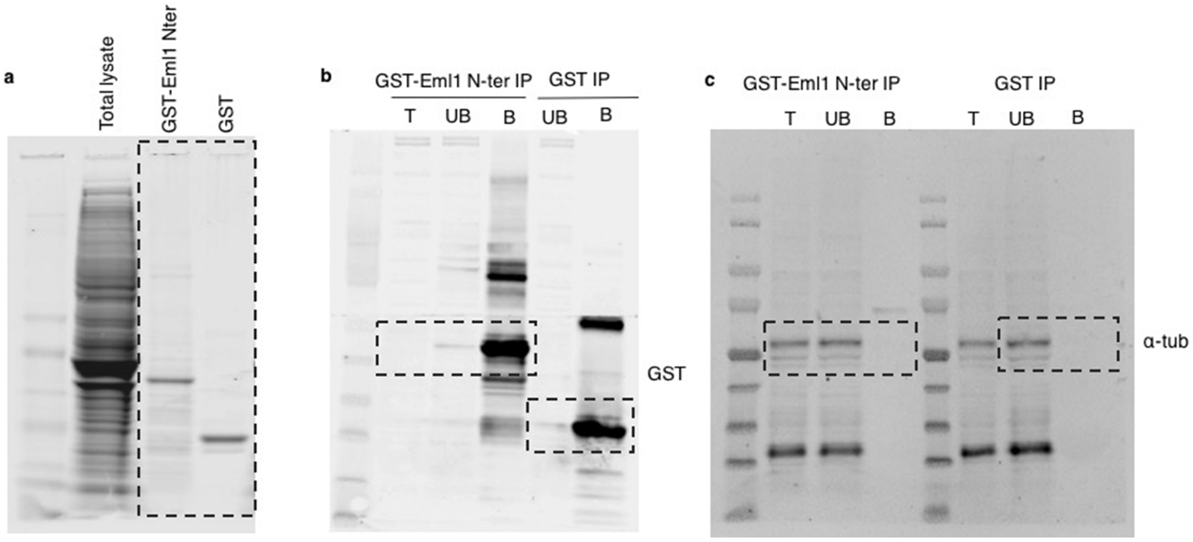

Supplementary Figure S5. **Full-length pictures of the gel and blots presented in Supplementary Fig. S3.** (**a**) gel in Supplementary Fig. S3a. (**b,c**) Original blots from which the bands shown in Supplementary Fig. S3b were cropped. The same IP samples were run twice on two different gels due to the necessity of detecting GST-Eml Nter and control GST (**b**) separately from alpha-tubulin (**c**) to avoid possible band overlap due to similar molecular weight of the proteins. Regions cropped in Supplementary Fig. S3 are indicated by boxed regions.
